# Prognostic value of lactate clearance, fluid balance, and APACHE II score in patients with cardiogenic shock receiving extracorporeal membrane oxygenation

**DOI:** 10.3389/fcvm.2025.1557909

**Published:** 2025-05-07

**Authors:** Qi-Feng Zhang, Shuang-Long Zhang, Gang Li, Miao Guo, Xiao-Xia Qi, Xiao-Hui Xing, Zheng Wang

**Affiliations:** ^1^ Department of Critical Care Medicine, Peking University International Hospital, Beijing, China; ^2^ Department of Neurosurgery, Tianjin Medical University General Hospital, Tianjin, China

**Keywords:** cardiogenic shock, extracorporeal membrane oxygenation therapy, fluid balance, lactate clearance, APACHE II score

## Abstract

**Objective:**

The objective of this study is to examine the prognostic value of lactate clearance, fluid balance, and the Acute Physiology and Chronic Health Evaluation II (APACHE II) score in patients with cardiogenic shock undergoing treatment with extracorporeal membrane oxygenation (ECMO).

**Methods:**

A retrospective analysis was conducted on 32 patients with cardiogenic shock who underwent ECMO in the Intensive Care Unit of Peking University International Hospital between January 2021 and June 2024. The patients were categorized into a survival group (*n* = 14) and a non-survival group (*n* = 18) based on their clinical outcomes. Baseline characteristics, including age, sex, and body weight and so on were collected for both groups. Multivariate logistic regression analysis was used to identify risk factors influencing patient prognosis. The prognostic value of relevant indicators was assessed using receiver operating characteristic curve analysis, while Pearson's correlation analysis was conducted to assess the relationships between specific indicators.

**Results:**

Lactate clearance was significantly lower in the non-survival group compared to the survival group, while fluid balance and APACHE II scores were notably higher in the non-survival group (*p* < 0.05). Based on the predictive model, the APACHE II score demonstrated the highest specificity for prognosis at 97.4%, whereas the combined indices exhibited the highest sensitivity at 95.5%. Additionally, lactate clearance revealed a negative correlation with both fluid balance and APACHE II scores (*p* < 0.05).

**Conclusion:**

This pilot study demonstrated that lactate clearance, fluid balance, and APACHE II score are valuable prognostic indicators for patients; however, the predictive accuracy of individual indicators is limited. The combined assessment of these indices provides a more robust and reliable predictive performance.

## 1 Introduction

Cardiogenic shock is a clinical syndrome characterized by a sudden decrease in cardiac output and subsequent hypoperfusion of tissues and organs, posing a severe threat to patient health due to metabolic disturbances and damage to critical organs (1). The condition often leads to ischemic injury and necrosis of vital tissues and organs, necessitating timely and effective medical interventions. Advances in cardiopulmonary assist technologies have led to the development of extracorporeal membrane oxygenation (ECMO), which provides vital support for the management of cardiogenic shock disease (2). ECMO functions by delivering cardiopulmonary support through extracorporeal medical devices, providing potential improvement in clinical outcomes for patients who are critically ill (3).

However, clinical experience highlights significant variability among patients with cardiogenic shock, where prognosis is influenced by multiple factors (4). Thus, it is essential to adopt not only scientifically validated interventions but also comprehensive prognostic assessments to optimize outcomes and meet the personalized treatment needs of patients.

Lactate levels, a common marker of tissue perfusion, are significantly elevated during disease states, and lactate clearance serves as an important indicator of metabolic efficiency and disease severity. Fluid balance reflects the fluid retention and load of the body, while the APACHE II score provides a standardized measure of disease severity. This study posits that these three parameters hold significant value in prognostic evaluation. Exploring their predictive efficacy is, therefore, of substantial clinical and research importance.

## 2 General data and method

### 2.1 General data

A retrospective analysis was conducted on 32 patients with cardiogenic shock who received ECMO in the Intensive Care Unit of Peking University International Hospital. These patients were categorized into a survival group (*n* = 14) and a non-survival group (*n* = 18) based on their clinical outcomes. A comparison of baseline characteristics is presented in Table 1.

**Table 1:** Comparison of general data of patients (n, %), ($\bar{x}$±s).

Index		Survival group (*n* = 14)	Non-survival group (*n* = 18)	*χ^2^*/*t*	*P*
Sex	Male	8 (57.14)	12 (66.66)	0.287	0.592
	Female	6 (42.85)	6 (33.33)		
Age (years)		72.83 ± 6.11	73.45 ± 6.02	0.381	0.705
Body weight (kg/m^2^)		26.23 ± 2.44	26.15 ± 2.36	0.124	0.902
Blood potassium (mmol/L)		4.62 ± 1.22	4.68 ± 1.35	0.177	0.860
Blood sodium (mmol/L)		140.35 ± 6.38	141.22 ± 6.51	0.505	0.615
Prothrombin activity (%)		62.83 ± 11.57	64.32 ± 12.37	0.469	0.641
Sequential Organ Failure Assessment (points)		15.35 ± 3.68	15.88 ± 3.95	0.523	0.603
pH value		7.28 ± 0.33	7.34 ± 0.32	0.686	0.495
Lactate clearance (%)		39.19 ± 14.66	19.13 ± 10.13	5.672	<0.001
Fluid balance (ml/kg)		20.73 ± 6.34	25.66 ± 6.01	2.957	0.004
APACHE II score (points)		10.47 ± 2.80	15.04 ± 3.13	5.834	<0.001

### 2.2 Inclusion and exclusion criteria

Inclusion Criteria: (1) Patients meeting the diagnostic criteria for cardiogenic shock (5); (2) Diagnosis of cardiogenic shock confirmed by echocardiography and microcirculatory perfusion assessments; (3) Patients with low tissue perfusion; (4) Ineffectiveness of fluid resuscitation and blood volume supplementation, necessitating ECMO; and (5) Cardiogenic shock primarily caused by underlying heart disease.

Exclusion Criteria: (1) Recent treatment with nitroprusside or metformin; (2) Presence of other malignant tumors, severely impaired renal function, metabolic disorders, significant traumatic brain injury, cerebral hemorrhage, or drug intoxication; (3) Decision by the family to give up active treatment.

### 2.3 Methods

(1) Preparation for ECMO: The ECMO equipment and associated pipelines were coated with heparin. Percutaneous punctures of the femoral artery and femoral vein were conducted sequentially, followed by the insertion of an 18F arterial drainage cannula and an 18F venous drainage cannula. The veno-arterial (V-A) mode was selected for the procedure.
(2) Operational Management: During the ECMO procedure, arterial blood gas levels of the patient were monitored every six hours to ensure that arterial blood oxygen saturation remains above 95%. Unfractionated heparin was administered as an anticoagulant, with dosage adjustments made, based on whole blood activated coagulation time. Pump flow, ventilation strategies, and vasoactive drug regimens were adjusted in response to changes in blood oxygenation and hemodynamic parameters.
(3) Weaning from ECMO: Weaning was initiated when the ECMO flow was < 10% of cardiac output, blood pressure > 80 mmHg with a pulse pressure > 20 mmHg after discontinuation of vasoactive drugs, and vital signs remained stable for a period exceeding six hours. These conditions were mandatory to proceed with weaning.

Therapeutic goals were guided by clinical protocols for managing cardiogenic shock and individualized physician assessments. Institutional guidelines set targets of systolic blood pressure ≥90 mmHg and a cardiac index ≥2.2 L/min/m^2^ to optimize tissue perfusion and organ function during ECMO support.

### 2.4 Survey tools and data collection

Upon admission, data collected included age, sex, body weight, serum potassium, serum sodium, prothrombin activity, Sequential Organ Failure Assessment (SOFA) score, potential of hydrogen (pH), lactate clearance, fluid balance, and APACHE II score. Among them, serum potassium and sodium levels were measured using electrolyte analysis, with normal reference ranges of 3.5–5.5 mmol/L and 135–145 mmol/L, respectively. Coagulation was assessed through prothrombin activity measurement, with a normal range of 70%–130%. The SOFA score was analyzed, with a total scoring range of 0–24 points (6). Arterial blood samples were analyzed using a blood gas biochemistry analyzer to determine pH levels and lactate clearance. The normal pH range was 6.9–7.7. Lactate clearance was calculated using the formula: (initial value—retest value)/initial value * 100%. The formula for calculating the liquid balance is (total liquid input—total output for 24 h on the machine)/body weight. The APACHE II score incorporated factors such as age, type of surgery, function of vital organs, and physiological abnormalities, with scoring based on clinical presentation (7).

### 2.5 Statistical analysis

Statistical analysis was conducted using SPSS version 24.0. Measurement data with a normal distribution, including age, body weight, serum potassium, serum sodium, prothrombin activity, SOFA score, pH value, lactate clearance, fluid balance, and APACHE II score, are expressed as mean ± standard deviation, and comparisons were conducted using the *t*-test. Categorical data, such as sex, are presented as percentages (%), with comparisons made using the chi-squared test. A *post-hoc* power analysis was performed using GPower software, indicating that our sample size provided sufficient power for detecting significant differences.

Binary logistic regression analysis was used to identify independent prognostic risk factors in patients with cardiogenic shock treated with extracorporeal membrane oxygenation. ROC curve analysis was conducted using MedCalc version 22 to assess the predictive performance of the indicators. Pearson correlation analysis was applied to examine the relationships between relevant parameters. A *p*-value of <0.05 was considered statistically significant.

## 3 Results

### 3.1 Comparison of general data of patients

Lactate clearance was significantly lower in the non-survival group compared to the survival group, while fluid balance and APACHE II scores were significantly higher in the non-survival group (*p* < 0.05). No statistically significant differences were observed in other relevant indices between the two groups (*p* > 0.05) (Table 1).

### 3.2 Multivariate logistic regression analysis of the prognosis

Based on multivariate analysis, the APACHE II score, lactate clearance, and fluid balance were identified as independent risk factors for patients with cardiogenic shock undergoing extracorporeal membrane oxygenation (*p* < 0.05) (Table 2).

**Table 2:** Prognostic multivariate logistic regression analysis.

Variables	*B-*value	Standard error	Wald	*p-*value	95% CI	
					Lower limit	Upper limit
Lactate clearance (%)	−0.206	0.074	7.827	0.005	0.705	0.940
Fluid balance (ml/kg)	0.282	0.124	5.142	0.023	1.039	1.692
APACHE II score (points)	0.917	0.330	7.721	0.005	1.310	4.778
Constant	−13.557	5.471	6.141	0.013	-	

### 3.3 Analysis of the prognostic value of APACHE Ⅱ score, lactate clearance and fluid balance

**Figure 1:**
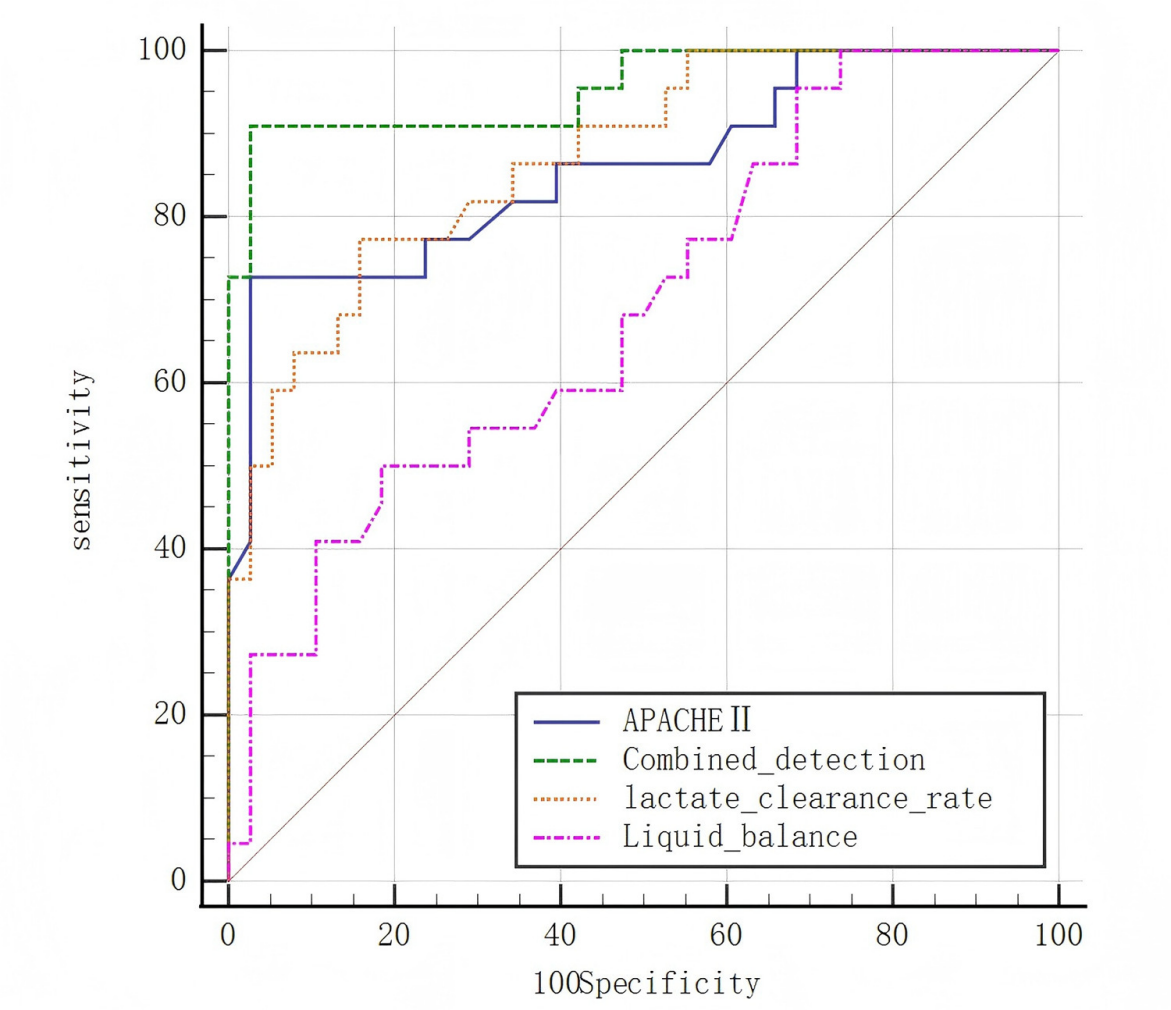
Receiver operating characteristic (ROC) curves for the prognostic prediction of cardiogenic shock outcomes based on lactate clearance, fluid balance, and APACHE II score. The combined model exhibited the highest predictive sensitivity.

The ROC curve analysis demonstrated that the APACHE II score exhibited the highest prognostic prediction specificity at 97.4%. Additionally, the combined detection approach achieved a significantly higher sensitivity of 95.5% compared to the use of single indices (*p* < 0.05) (Table 3 and Figure 1).

**Table 3:** Examination of the APACHE II score, lactate clearance, and fluid balance prognostic values.

Variables	AUC	SE	95%CI	Sensitivity	Specificity
Lactate clearance (%)	0.871	0.0462	0.760∼0.944	77.3	84.2
Fluid balance (ml/kg)	0.690	0.0710	0.557∼0.803	50.0	81.6
APACHE II score (points)	0.860	0.0540	0.746∼0.936	72.7	97.4
Combined detection	0.980	0.0145	0.905∼0.999	95.5	94.7

### 3.4 Correlation analysis of APACHE Ⅱ score, lactate clearance, and fluid balance

Pearson's correlation analysis revealed a negative correlation between lactate clearance and both fluid balance and the APACHE II score (*p* < 0.05) (Table 4).

**Table 4:** Analysis of the correlation between fluid balance, lactate clearance, and APACHE II score.

Item	Lactate clearance (%)	Fluid balance (ml/kg)	APACHE II score (points)
Lactate clearance (%)	1	−0.186	−0.276^a^
Fluid balance (ml/kg)		1	0.164
APACHE II score (points)			1

^a^ indicates that the correlation is significant at the 0.05 level (two-tailed).

### 3.5 Subgroup analysis using cox regression model

**Figure 2:**
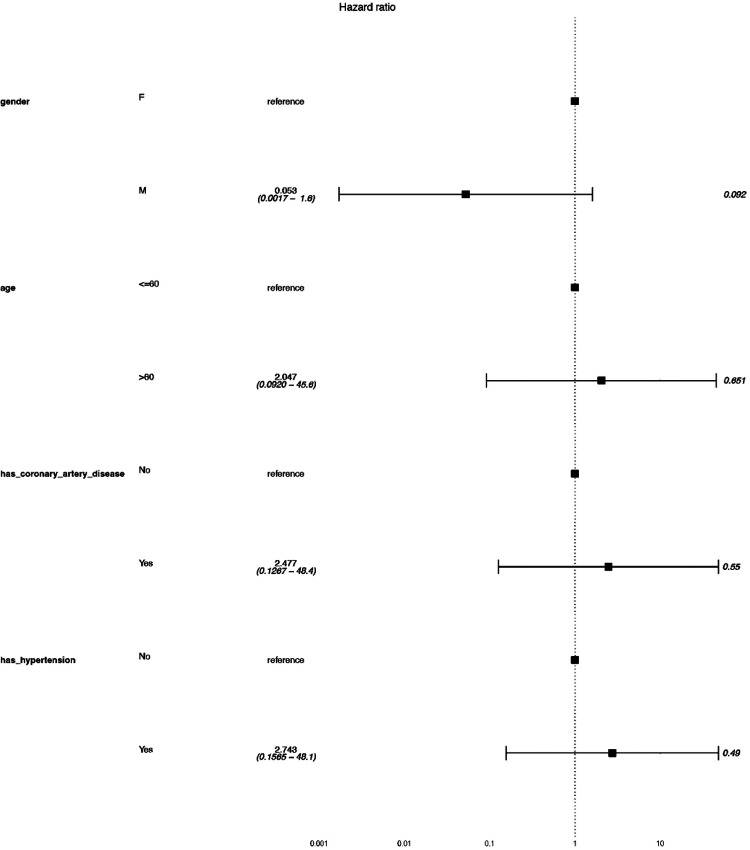
Subgroup analysis results using cox regression model.

Exploratory subgroup analysis revealed that hypertensive patients exhibited a hazard ratio of 2.74 (95% CI: 0.156–48.1), though statistical significance was not achieved (*p*-value = 0.368). Patients with coronary artery disease showed no significant prognostic differences compared to those without (Figure 2). Comorbidities such as coronary artery disease and hypertension were recorded, and their specific impact on outcomes was analyzed using cox regression model demonstrated in the subgroup analysis. The results suggested that the two comorbidities did not influence the survival in our data.

## 4 Discussion

Cardiogenic shock, characterized by multi-organ dysfunction resulting from a sudden decrease in cardiac output and reduced tissue perfusion, is associated with rapid onset and poor prognosis (8, 9). In the early stages, the disease commonly presents with hypotension and impaired consciousness, which can progress to loss of consciousness and adverse outcomes if left untreated (10). ECMO is recognized as an effective therapeutic intervention for such patients. However, clinical observations have highlighted that prognosis is influenced by multiple factors, necessitating a focus on the development of comprehensive prognostic assessment methods (11).

The findings of this study indicate that lactate clearance was significantly lower in the non-survival group compared to the survival group, while fluid balance and APACHE II scores were notably higher in the non-survival group (*p* < 0.05). Moreover, lactate clearance, fluid balance, and APACHE II score were identified as independent prognostic risk factors for patients with cardiogenic shock undergoing ECMO (*p* < 0.05). These results align with those reported by Li et al., and the following mechanisms may explain these findings (12). While individual indicators have been studied previously, our work uniquely integrates lactate clearance, fluid balance, and APACHE II score to provide a comprehensive prognostic model, demonstrating improved predictive accuracy.

Patients with cardiogenic shock often exhibit impaired tissue perfusion, leading to microcirculatory disturbances. Arterial blood lactate levels serve as a sensitive marker for tissue ischemia and hypoxia, reflecting the adequacy of oxygen supply and perfusion. Although a single lactate measurement provides useful insights, dynamic changes in lactate levels offer greater prognostic value. In this study, lactate clearance was used as a prognostic indicator. Higher lactate clearance levels were associated with improved responsiveness to ECMO therapy and significant alleviation of ischemic and hypoxic conditions. Patients in the survival group demonstrated effective circulation under ECMO support, ensuring adequate perfusion of vital organs and enhancing lactate clearance efficiency. Conversely, patients in the non-survival group experienced excessive ventricular preload, severe tissue ischemia, and persistent elevated arterial lactate levels, which were not effectively resolved, ultimately leading to unfavorable outcomes.

Fluid balance is another critical factor influencing prognosis in patients who are critically ill. In the initial stages of treatment, patients with cardiogenic shock often require substantial fluid resuscitation to maintain cardiopulmonary bypass. However, these patients are at risk of developing acute kidney injury due to volume depletion, which can result in fluid accumulation. In the survival group, effective ECMO therapy stabilized cardiopulmonary bypass, alleviated renal dysfunction, and restored fluid balance. In contrast, the non-survival group experienced persistent systemic circulatory and renal dysfunction, leading to fluid imbalance and poor outcomes.

The APACHE II score, a widely used measure of disease severity in patients who are critically ill, was positively correlated with prognosis.

The findings of this study revealed that the APACHE II score demonstrated the highest predictive specificity at 97.4%, while the combined indices exhibited the highest predictive sensitivity at 95.5% and a specificity of 94.7%. The use of a single index for prognosis is influenced by individual variability within the patient population, whereas a combined evaluation approach effectively mitigates this limitation, enabling a more comprehensive assessment of patient outcomes.

Additionally, a negative correlation between lactate clearance and both fluid balance and APACHE II score (*p* < 0.05) was identified. Lactate serves as a critical marker for tissue and organ hypoxia, with lactate levels positively correlating with disease severity. Lactate clearance reflects the resolution of lactate accumulation in the patient's circulatory system. During the initial stages of treatment, blood lactate levels are typically elevated. In patients who respond favorably to ECMO therapy, lactate accumulation progressively decreases, resulting in a high lactate clearance rate. Conversely, patients experiencing prolonged fluid retention and critical diseases exhibit impaired lactate clearance, characterized by a high APACHE II score, elevated fluid balance, and low lactate clearance levels.

However, this study has several limitations. Firstly, data on the presence of underlying comorbidities were not collected, making it unclear whether these conditions influenced patient prognosis. Despite rigorous statistical analysis, some confounding variables such as medication history and pre-existing conditions may have influenced the results. Subgroup analysis suggests hypertension may influence prognosis, though further validation is needed due to wide confidence intervals. Future studies with larger cohorts should explore these relationships more robustly. Second, the study population consisted of patients with relatively similar age profiles, limiting the ability to explore the prognostic differences between younger, middle-aged, and older adult patients. Finally, the small sample size may have introduced statistical bias, potentially leading to missing or inconclusive data. Another key limitation of this study is its retrospective nature, which may introduce selection bias. Additionally, the small sample size may affect the statistical robustness of our findings. Future studies with larger sample sizes and prospective validation are warranted. The study also did not comprehensively analyze the influence of pre-existing comorbidities due to lack of data, which could have affected patient outcomes. Central venous oxygen saturation data were also not included. Future studies should focus on addressing these limitations by incorporating a larger and more diverse sample size, accounting for the presence of underlying comorbidities, and examining prognostic variations across different age groups. Such efforts will enable more comprehensive findings and contribute to the advancement of high-quality medical services.

In conclusion, this pilot study demonstrates that lactate clearance, fluid balance, and the APACHE II score are valuable indicators for prognostic evaluation in patients with cardiogenic shock undergoing ECMO. Furthermore, the combined assessment of these indices exhibited superior efficacy compared to single-index evaluations. The findings presented here highlight the need for a more comprehensive investigation into the role of lactate clearance, fluid balance, and the APACHE II score in guiding clinical decision-making.

## Data Availability

The raw data supporting the conclusions of this article will be made available by the authors, without undue reservation.
